# Was It Extra-Uterine Pregnancy?

**Published:** 1889-12

**Authors:** E. Cross

**Affiliations:** San Antonio, Texas


### WAS IT EXTRA-UTERINE PREGNANCY?

DR. CROSS ON DR. LOWRY'S CASE.

SAN ANTONIO, TEXAS, NOV. 10, 1889.

*Editor Daniel's Texas Medical Journal:*

Through the columns of your esteemed JOURNAL, I wish to review briefly, a case reported in the September number of the

Texas Courier-Record, by Dr. Lowry, of this city, entitled, "A case of extra-uterine pregnancy terminating naturally."

The whole history of this case, as reported, is truly phenomenal, and it is to be regretted that the reporter has not gone more into detail, as many points of interest are left unexplained. In reviewing this report, the first point of interest which attracts attention, is the remarkable diagnostic powers of the reporter, in that he should recognize the presence of ectopic pregnancy, or any other form of extra-uterine tumor, without having one symptom which pointed to the existence of such a condition.

A patient approaching the menopause calls upon him, (Dr. L.), complaining of the ordinary reflex symptoms of early pregnancy, viz: nausea, morning sickness, etc. The patient "a large corpulent one, with large deposits of adipose tissue in the abdominal walls," is examined once with a sound, by palpation and with a speculum. The sound being "introduced very gently," shows the cavity of the womb to be only two and one half inches in depth; by palpation nothing is discovered, but from the examination with the speculum, the reporter is convinced of the existence of an extra-uterine tumor. Would not the profession be under obligations to the Doctor if he would give us one indication he found to base his diagnosis upon? Certain it is the sound negatived the existence of any form of uterine tumor, as two and a half inches (which is the depth claimed of this womb) is below the average measurement in a nullipara, and it is the experience of the profession, that the womb is promptly increased in size either from extra uterine pregnancy, or other uterine tumors. As to what special condition was revealed by the speculum which led to so prompt a diagnosis, we are not informed. The patient is told that she is in a critical condition. "That she has a tumor; and an extra-uterine pregnancy is suspected." With a request to call again she is discharged, and not seen nor heard from for seven months, when the reporter is called in haste to find his patient in labor, "the membranes having given way and a profuse discharge of amniotic fluid having escaped." How unlike the usual course of ectopic pregnancy this history! No untoward symptom has developed, no inward pains during the full term; and is it not presumable that the patient, after having been warned of her condition, would have been on the alert for any unusual development? None are reported. After the waters are discharged a large irregular tumor is outlined "lying on the right of, yet closely attached to the womb." No digital examination is yet made, as the os is not sufficiently patulous to admit the finger, but a "large blunt sound is introduced, coming firmly against the fundus, at five inches;" the sound now "by a slight deflection to the right" (tumor on same side), is passed nine inches into, and through the right fallopian tube. Now the reporter is convinced he has an extra-uterine pregnancy to deal with, and feels that "something must be done," yet wisely does nothing. Three long wearisome days have passed,—expectant ones. The uterus having expelled a portion of its contents, rests; another examination is deemed necessary; (though no urgent symptons have appeared); the os is found sufficiently open to permit the finger; now, through the vagina it passes; nothing unusual there—thence through the os into the uterus, and there the extra-uterine tumor is found. The finger (as stated) "coming in contact with the caput succedaneum." A strange place to find an ectopic pregnancy, but there it was—a few pains expel it, "a living female infant, per via naturalis." The placenta does not follow immediately, and the hand is now introduced into the organ that three days previously only admitted a sound, and whose depth was five inches only—for the purpose of removing the placenta, which was found adherent to some surface (what or where the reporter fails to tell us); but after passing the hand into the womb he found an opening to the right; through something into something, where the placenta is found and removed through the vagina. No hemorrhage follows; indeed no untoward symptom. The patient is discharged after the ninth day well; and thus ends the case as reported.

Naturally the following queries would arise: What form of ectopic pregnancy could have existed with such a history and termination (normal in all respects)? Not fallopian, as the tube was empty; not ventral, in any of its forms,—for, if so, how did the fœtus get into the uterine cavity? unless, like a chicken confined in its shell, it becomes wearied of its long confinement, and instead of picking its way into the womb, scratched it; though this theory is untenable; as how could it be possible to pass a sound through the womb into the right fallopian tube, with a large tumor, (a female baby,) closely attached to the same side? It was not ovarian; nor could it be that there existed a double uterus; that sound passed into the right fallopian tube. Then was it not a case of mistaken diagnosis? Yea, a normal gestation, with hour glass contractions of the uterus, as the conditions were favorable for the production of irregular contractions of the womb, viz: a premature escape of the waters; and a retained placenta. With a hope to have some explanation offered as to the location of the placenta, and the form of extra-uterine pregnancy that existed in Dr. L's case, I am sir, respectfully yours,

E. CROSS.

